# Numerical Simulation of Boundary-Driven Acoustic Streaming in Microfluidic Channels with Circular Cross-Sections

**DOI:** 10.3390/mi11030240

**Published:** 2020-02-26

**Authors:** Junjun Lei, Feng Cheng, Kemin Li

**Affiliations:** 1 State Key Laboratory of Precision Electronic Manufacturing Technology and Equipment, Guangdong University of Technology, Guangzhou 510006, China; cff951151311@163.com (F.C.); lkm1300454596@163.com (K.L.); 2 Guangzhou Key Laboratory of Non-traditional Manufacturing Technology and Equipment, Guangdong University of Technology, Guangzhou 510006, China

**Keywords:** acoustic streaming, boundary-driven streaming, cylindrical channel, limiting velocity method, acoustofluidics, microfluidics, micromanipulation

## Abstract

While acoustic streaming patterns in microfluidic channels with rectangular cross-sections have been widely shown in the literature, boundary-driven streaming fields in non-rectangular channels have not been well studied. In this paper, a two-dimensional numerical model was developed to simulate the boundary-driven streaming fields on cross-sections of cylindrical fluid channels. Firstly, the linear acoustic pressure fields at the resonant frequencies were solved from the Helmholtz equation. Subsequently, the outer boundary-driven streaming fields in the bulk of fluid were modelled while using Nyborg’s limiting velocity method, of which the limiting velocity equations were extended to be applicable for cylindrical surfaces in this work. In particular, acoustic streaming fields in the primary (1, 0) mode were presented. The results are expected to be valuable to the study of basic physical aspects of microparticle acoustophoresis in microfluidic channels with circular cross-sections and the design of acoustofluidic devices for micromanipulation.

## 1. Introduction

Ultrasonic particle manipulation (UMP) is a contactless method that is well-suited for micromanipulation in microfluidic systems. Most UPM devices that are shown in literature use standing waves to manipulate particles for applications, such as patterning [1,2,3,4], focusing [5,6,7], and separation [8,9,10] of microparticles. When a standing wave field is established in a microfluidic channel, the movements of particles suspended in the fluid medium are determined by two main forces, i.e., the acoustic radiation force (ARF) and the acoustic streaming (AS) induced drag force, which scale with the volume and diameter of the particle, respectively. In most UPM devices, ARF is the main engine for particle manipulation, while the AS effects are mostly regarded as disturbances as they usually place a lower limit on the particle size that can be manipulated by the ARFs [11]. In general, the two main forces could balance when the particle size reaches a certain value, which is, for example, approximately 1.6 μm at a frequency of 1 MHz [12]. However, structured AS vortices have also been designed to bring particles to desired positions for many microfluidic applications [13,14,15].

The AS field in a microfluidic channel of particular interest is generally dominated by boundary-driven streaming, which arises from the absorption of acoustic momentum flux in the viscous boundary layer [16]. Another type of well-known streaming pattern, the Eckart streaming [17], generally requires acoustic absorption over a longer distance, e.g., multi-wavelengths, than those that are typically found in microfluidic channels [18]. The journey of boundary-driven streaming theory started from Rayleigh [19], who derived equations for the AS field between two infinite walls outside the viscous boundary layer, which is, thus, nowadays commonly referred to as ‘outer streaming’ or ‘Rayleigh streaming’. Schlichting [20] later found that each outer streaming vortex is associated with a vortex confined to the boundary layer, being known as ‘inner streaming’ or ‘Schlichting streaming’. An analytical solution for solving both the inner and outer streaming fields that were generated by an one-dimensional standing wave in rectangular channels were derived by Hamilton et al. [21], who showed that inner and outer streaming vortices co-exist in wide channels, while outer streaming vortices could disappear in sufficiently narrow channels. Nyborg [22] found that, in wide channels, the AS velocity at the extremity of the inner vortex (the limiting velocity (LV)) can be approximated as a function of the linear acoustic velocity field and the outer streaming can be effectively predicted by taking the LV as a slip boundary condition. With the rapid progress of experimental and computational techniques, recent experimental [23] and modelling [24] works have shown excellent agreement with predictions that are based on these well-established theories.

New boundary-driven streaming patterns that cannot be explained by Rayleigh’s theory have been observed in the bulk of fluid in addition to the classical Rayleigh streaming whose orientations are generally perpendicular to the driving boundaries and the transducer radiating surfaces in experimental UPM devices. Typically, in thin-layer UPM devices with high aspect-ratio rectangular fluid channels, AS vortices with orientations parallel to the driving boundaries and the transducer radiating surfaces, thus called ‘transducer-plane streaming’, have been experimentally observed [24,25,26]. Through three-dimensional (3D) numerical simulations while using Nyborg’s limiting velocity method (LVM), it was found that they were closely related to the local active intensity fields, which tend to circulate when the local acoustic field is a superposition of certain standing and travelling wave components [27,28].

While boundary-driven streaming fields in microfluidic channels with rectangular cross-sections have been extensively studied in literature, less attention has been paid to those in microfluidic channels with non-flat boundaries [29]. In this work, we present numerical simulations of boundary-driven streaming on cross-sections of a cylindrical cavity in a square glass capillary, which has been recently used for two-dimensional (2D) focusing of microparticles [30]. Particularly, Nyborg’s LV equations have been extended for predicting outer streaming fields in cylindrical fluid channels.

## 2. Theory of Acoustic Streaming

In the following, we use bold fonts to represent vectors to distinguish them from scalar quantities described with normal-emphasis fonts. The derivation of basic acoustic and streaming equations has been widely presented in literature. For a homogeneous isotropic fluid, the continuity and momentum equations for the fluid motion are [31]

(1) $$\frac{\partial \rho }{\partial t}+\nabla \cdot \left(\rho u\right)=0,$$

(2) $$\rho \left(\frac{\partial u}{\partial t}+u\cdot \nabla u\right)=-\nabla p+\mu \nabla^{2}u+\left(\mu_{b}+\frac{1}{3}\mu \right)\nabla \nabla \cdot u,$$

where ρ is the fluid density, t is time, u is the fluid velocity, p is the pressure, and μ and $\mu_{b}$ are the dynamic and bulk viscosity coefficients of the fluid, respectively.

While using the perturbation theory [32,33], the fluid density ρ, pressure p, and velocity u are expressed as

(3) $$\rho =\rho_{0}+\rho_{1}+\rho_{2}+\cdots ,$$

(4) $$p=p_{0}+p_{1}+p_{2}+\cdots ,$$

(5) $$u=u_{1}+u_{2}+\cdots ,$$

where the subscripts 0, 1, and 2 represent the static (absence of ultrasonic excitation), first-order, and second-order quantities, respectively.

Substituting Equations (3)–(5) into Equations (1) and (2) and while taking the first-order into account, Equations (1) and (2) become

(6) $$\frac{\partial \rho_{1}}{\partial t}+\rho_{0}\nabla \cdot u_{1}=0,$$

(7) $$\rho_{0}\frac{\partial u_{1}}{\partial t}=-\nabla p_{1}+\mu \nabla^{2}u_{1}+\left(\mu_{b}+\frac{1}{3}\mu \right)\nabla \nabla \cdot u_{1}.$$

Repeating the above procedure, while considering the first- and second-order, and taking the time average of Equations (1) and (2), the continuity and momentum equations are then turned into

(8) $$\nabla \cdot \bar{\rho_{1}u_{1}}+\rho_{0}\nabla \cdot \bar{u_{2}}=0,$$

(9) $$-\rho_{0}\bar{u_{1}\nabla \cdot u_{1}+u_{1}\cdot \nabla u_{1}}=-\nabla \bar{p_{2}}+\mu \nabla^{2}\bar{u_{2}}+\left(\mu_{b}+\frac{1}{3}\mu \right)\nabla \nabla \cdot \bar{u_{2}},$$

where the upper bar $\bar{\cdot }$ indicates the time average of the quantity below.

In most UPM devices where the dimensions of the fluid channel are much larger than the boundary layer thickness and, thus, only the outer streaming fields are usually of interest, the 2D or 3D boundary-driven streaming fields in the bulk of the fluid channel can be effectively predicted while using Nyborg’s LVM. In such models, Equations (8) and (9) can be further simplified to [34]

(10) $$\nabla \cdot \bar{u_{2}}=0,$$

(11) $$-\nabla \bar{p_{2}}+\mu \nabla^{2}\bar{u_{2}}=0.$$

## 3. Numerical Model

**Figure 1:**
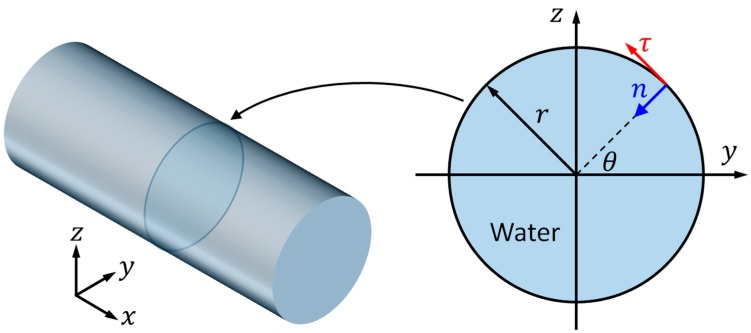
A two-dimensional reduced-fluid model (**right**), which takes a cross-section of a cylindrical cavity (**left**), was considered. r is the radius of the cross-section of the channel; θ is the angle of a point on the boundary; and, τ and n represent the tangential and inward normal directions of the point on the boundary, respectively.

Here, we consider a cylindrical fluid channel with radius r=0.45 mm (the size of fluid channel recently reported for particle manipulation [30]), where the theoretical resonant frequency of first resonant mode, the (1, 0) mode, is around 1 MHz. In this work, for numerical efficiency, a 2D reduced-fluid model representing a cross-section of the cylindrical cavity, as shown in Figure 1, was considered and Nyborg’s LVM was applied to simulate the outer streaming fields in the bulk of the fluid. Reduced-fluid models and the LVM have been widely used for predicting boundary-driven streaming fields in UPM devices [25].

### 3.1. Extention of the Limiting Velocity Method

Nyborg’s LVM, which derives two tangential LV components on a 2D vibrating surface, is valid if the radius of curvature of the surface is large when compared to the thickness of boundary layer [22]. It was later modified by Lee and Wang to a more generalized version [35]. In 3D xyz Cartesian coordinates, for example, on a planar surface normal to z, the two tangential LV equations are [25]

(12) uL=−14ωRe{u1du1*dx+v1du1*dy+u1*[(2+i)∇·u1−(2+3i)dw1dz]},

(13) vL=−14ωRe{u1dv1*dx+v1dv1*dy+v1*[(2+i)∇·u1−(2+3i)dw1dz]},

where ω is the angular frequency, Re{·} represents the real part of the quantity inside, $u_{1}$, $v_{1}$, and $w_{1}$ are the x, y, and z components of the first-order acoustic velocity vector, $u_{1}$, and the superscript, *, represents the complex conjugate. We assume here that the boundary of the circular domain is a summation of infinite number of segments, of which each could be approximated to a tiny straight line (or a single point), where the LVM might be applicable, to make this method valid to the case presented in this work.

As described in the right part of Figure 1, the position of each point on the boundary can be described by the radius r and an angle θ, as calculated from

(14) $$\theta =\tan^{-1}\left(\frac{z}{y}\right).$$

Additionally, the unit vectors in the tangential (τ) and inward normal (n) directions (Figure 1) of a boundary point can be expressed as

(15) $$\hat{\tau }=-\hat{y}\sin\theta +\hat{z}\cos\theta ,$$

(16) $$\hat{n}=-\hat{y}\cos\theta -\hat{z}\sin\theta ,$$

where $\hat{\cdot }$ represents the unit vector in the direction below. Accordingly, the tangential and normal components of the first-order acoustic velocity, $v_{1\tau }$ and $w_{1n}$, at a given point and their gradients in the tangential and normal directions, can be expressed as

(17) $$v_{1\tau }=-v_{1}\sin\theta +w_{1}\cos\theta ,$$

(18) $$w_{1n}=-v_{1}\cos\theta -w_{1}\sin\theta ,$$

(19) $$\frac{dv_{1\tau }}{d\tau }=-\frac{dv_{1\tau }}{dy}\sin\theta +\frac{dv_{1\tau }}{dz}\cos\theta ,$$

(20) $$\frac{dw_{1n}}{dn}=-\frac{dw_{1n}}{dy}\cos\theta -\frac{dw_{1n}}{dz}\sin\theta .$$

In this 2D model, the outer streaming fields in the bulk of the fluid are only driven by the tangential component LV, which is

(21) vLτ=−14ωRe{v1τdv1τ*dτ+v1τ*[(2+i)(dv1τdτ+dw1ndn)−(2+3i)dw1ndn]}.

While combining Equations (14) and (17)–(21), the distribution of LV on the circular boundary of the fluid channel can be obtained.

### 3.2. Numerical Implementations

The numerical simulations were implemented in the finite element software COMSOL [36], run on a HP EliteBook 820G4 running Windows 10 (64-bit), which was equipped with 8 GB RAM and Intel (R) Core (TM) i7-7500U processor of clock frequency 2.7 GHz. It takes about 20 s to complete the steps below.

Firstly, a ‘*Pressure Acoustics, Frequency Domain*’ interface was applied to simulate the first-order acoustic pressure field $p_{1}$ under different excitations (shown below) from the Helmholtz equation

(22) $$\nabla^{2}p_{1}+\frac{\omega^{2}}{c^{2}}p_{1}=0,$$

where c is sound speed in the fluid, and the first-order acoustic velocity vector field $u_{1}$ from the momentum conservation equation

(23) $$\rho_{0}\frac{\partial u_{1}}{\partial t}+\nabla p_{1}=0.$$

The resonant frequency was predicted from an *frequency sweep* study, which finds the frequency that gives the maximum $\bar{E_{ac}}$ (the average acoustic energy density in the model regime) [25].

Subsequently, to simulate the second-order acoustic streaming fields, a ‘*Laminar Flow*’ interface was used to solve Equations (10) and (11). The LV field that was derived from Equation (21) was applied to the circular boundary as a slip velocity boundary condition.

Table 1 shows the parameters used in simulations.

## 4. Results and Discussion

### 4.1. Boundary-Driven Acoustic Streaming in Rectangular Channels

**Figure 2:**
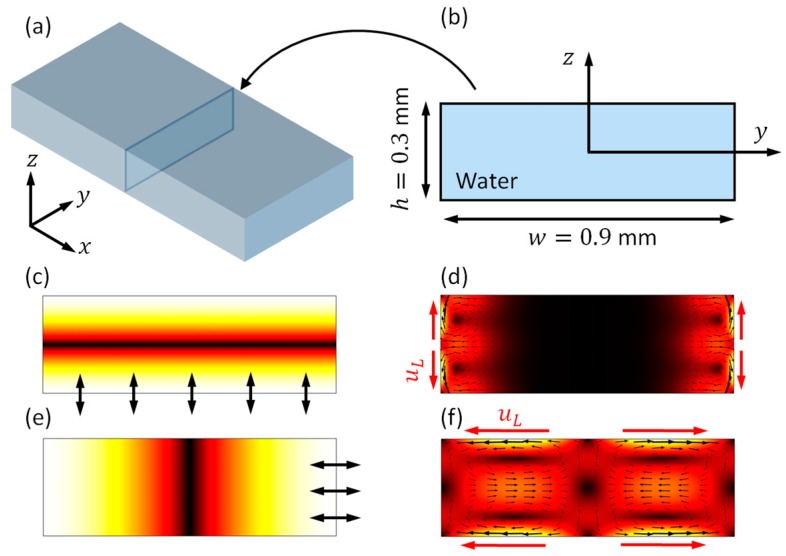
Numerical simulations of boundary-driven acoustic streaming fields in a fluid channel with rectangular cross-sections. (**a**) A typical three-dimensional (3D) rectangular fluid channel (where fluid goes in the x direction); (**b**) a two-dimensional (2D) reduced-fluid model representing a cross-section of (**a**); (**c**) a vertical half-wavelength standing wave field, generated by a harmonic vibration of the bottom boundary at f=2.469 MHz; (**d**) the outer streaming field for mode (**c**); (**e**) a lateral half-wavelength standing wave, generated by a harmonic vibration of the right boundary at f=0.823 MHz; and, (**f**) the corresponding outer streaming field in mode (**e**). Colors in (**c**–**f**) represent magnitudes (white for maximum and black for zero). $u_{L}$ is the limiting velocity.

Prior to demonstrating the results in the model that is shown in Figure 1, for comparison, the boundary-driven acoustic streaming fields in a fluid channel with a rectangular cross-section of similar size are introduced. As presented in Figure 2b, a 2D model with dimensions of 0.9 × 0.3 mm^2^, a cross-section of a 3D rectangular fluid channel (Figure 2a), was considered. With different driving conditions, vertical (Figure 2c) and lateral (Figure 2e) standing wave fields could be generated on cross-sections of the fluid channel, thus resulting in different outer streaming patterns. In these 2D models, as indicated with red arrows and $u_{L}$ in Figure 2d,e, the main limiting velocity fields for these two modes are working on different boundaries, thus generating two different outer streaming patterns on cross-sections of the fluid channel.

### 4.2. Boundary-Driven Acoustic Streaming in Circular Channels

In this section, numerical simulations of boundary-driven streaming patterns in the model described in Figure 1 were studied. As an example, here we present the outer streaming fields in the primary (1, 0) mode, which has been shown to be responsible for 2D microparticle focusing in a recent work [30].

#### 4.2.1. Mesh Size-Dependency Study

**Figure 3:**
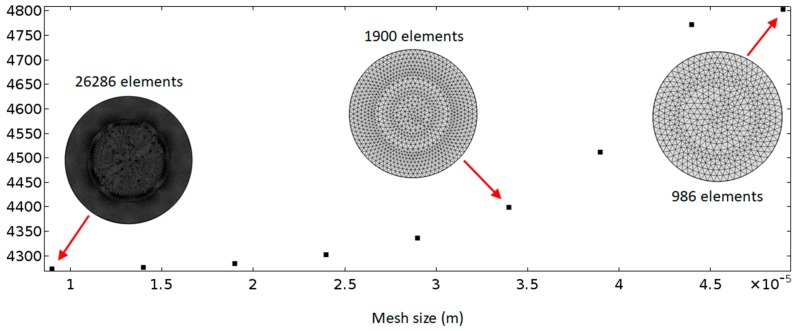
Mesh size-dependency studies. The vertical axis plots the average acoustic energy density (Pa) in the model regime.

As shown in the insets in Figure 3, a uniform distribution of triangular mesh elements was considered, because it is not necessary to resolve the boundary layer in this work. A mesh size-dependency study was conducted to determine the mesh size that is required for high accuracy to reduce the mesh-induced numerical error. Figure 3 plots the modelled average acoustic energy densities of the first mode in the whole fluid domain in models with different mesh sizes ranging from 9 μm to 49 μm. It can be seen that a stable solution could be achieved with a decrease of mesh size. For all the mesh sizes presented, a difference of ~12% on the average acoustic energy density was found between the two models with the smallest and the largest sizes, and a mesh size of ~40 µm could be used in simulations if allowed a 5% mesh-induced error. However, a maximum mesh size of 9 μm was used in the following simulations based on the high efficiency of this numerical model, to allow for modes that are more complex than the simple one explored in the dependency study (i.e., for modes higher than the basic (1, 0) mode) (it takes only ~20 s to complete all the acoustic and streaming simulations with this mesh size).

#### 4.2.2. Acoustic Pressure and Streaming Fields

**Figure 4:**
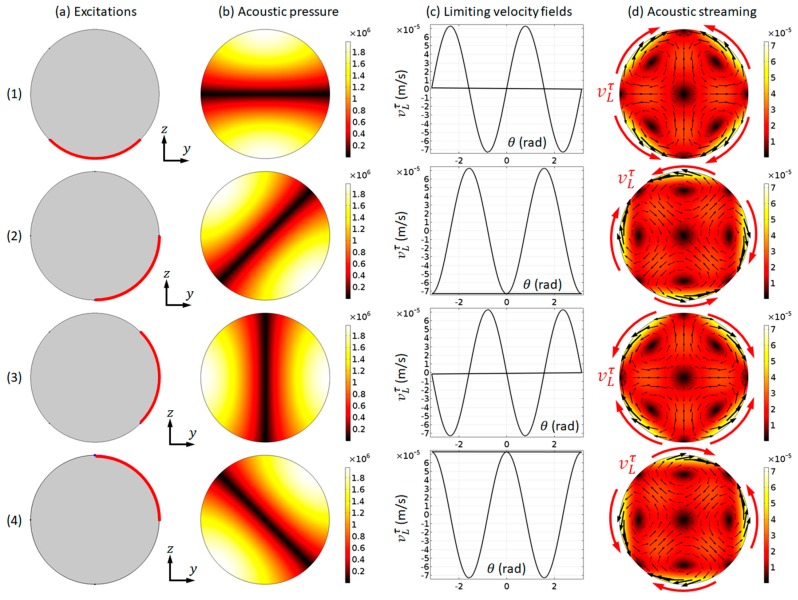
The modelling results for the first mode (f=0.9647 MHz). (**a**) Models with four different boundary vibrations (highlighted in red); (**b**) acoustic pressure magnitudes (Pa); (**c**) limiting velocity distributions; and, (**d**) acoustic streaming magnitudes (m/s) and vector (black arrows) fields. The magnitudes presented were obtained from a vibrating amplitude of 0.1 mm/s.

We choose to present the modelled acoustic pressure and streaming fields in the first mode here, i.e., the (1, 0) mode, to demonstrate the applicability of our numerical model described in Section 3 on the simulation of outer streaming fields in cylindrical channels. Figure 4 plots the modelled acoustic pressure, LV, and acoustic streaming fields in the first mode under four different boundary vibrations. It can be seen that, when compared to the corresponding patterns in a microfluidic channel with a rectangular cross-section (e.g., Figure 2), similarities and differences can both be seen. On the one hand, the main characteristic of the modelled outer streaming field is shown to be similar to the Rayleigh streaming in rectangular channels: (i) each streaming vortex occupies a quadrant of 2D fluid domain; and, (ii) on the driving boundaries, where the LVs are applied to the acoustic streaming velocities direct from acoustic pressure nodes to adjacent antinodes. On the other hand, the outer streaming patterns in a circular domain can be more diverse than those in a rectangular domain. It can be seen from Figure 2 that only two half-wavelength standing wave patterns (i.e., those shown in Figure 2c,e) (and thus two outer streaming patterns) could be generated in a fluid channel with rectangular cross-sections. However, as shown in Figure 4, four different (1, 0) modes were generated from four different boundary vibrations and, thus, four different outer streaming patterns were formed. They are ‘different’ patterns, because different trajectories would be seen if particles of streaming-dominated motions, e.g., nano-sized particles, were introduced in the fluid channel. Furthermore, the orientation of the single pressure nodal line could range from 0 to π if the full set of boundary vibrations were considered (only two axisymmetric boundary vibrations can result in a same acoustic pressure distribution). Different ultrasonic excitations can result in different vibrations of channel walls and, thus, theoretically, all of these different modes could be excited in a real experimental device.

#### 4.2.3. Trajectories of Microparticles

Here, we show here the trajectories of microparticles of different sizes driven by the two main forces, i.e., the acoustic radiation force $F_{r}$ and streaming-induced drag force $F_{d}$ to examine the effects of acoustic streaming on the microparticle acoustophoresis in a cylindrical channel, using (where the two main forces are calculated from the Gorkov [37] and Stokes equations, respectively)

(24) $$\frac{d}{dt}\left(m_{p}v\right)=F_{r}+F_{d},$$

(25) $$F_{r}=\nabla \left\{\frac{4\pi r^{3}}{3}\left[\frac{3\left(\rho_{p}-\rho_{f}\right)}{2\rho_{p}+\rho_{f}}\bar{E_{kin}}-\left(1-\frac{\rho_{f}c_{f}^{2}}{\rho_{p}c_{p}^{2}}\right)\bar{E_{pot}}\right]\right\},$$

(26) $$F_{d}=6\mu \pi r\left(u_{2}-v\right),$$

where $m_{p}$ is the particle mass, v is the particle velocity, r is particle radius, $\bar{E_{kin}}=0.25\rho_{f}{\left|u_{1}\right|}^{2}$ and $\bar{E_{pot}}={\left|p_{1}\right|}^{2}/\left(4\rho_{f}c_{f}^{2}\right)$ are the time-averaged kinematic and potential energy density, $\rho_{p}=1155\;\mathrm{kg}/m^{3}$ and $\rho_{f}=999.6\;\mathrm{kg}/m^{3}$ are the density of the particle and fluid, and $c_{p}=1962\;m/s$ and $c_{f}=1481.4\;m/s$ are the sound speed in particle and fluid.

**Figure 5:**
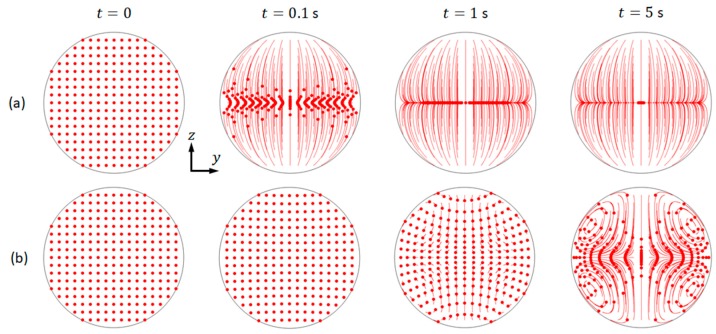
The modelled trajectories of microparticles under the combination of acoustic radiation and streaming-induced drag forces. Particles with diameters of (**a**) 10 µm; and, (**b**) 1 µm, where the red dots and the associated lines represent the microparticles and their trajectories, respectively.

As examples, it is presented the trajectories of microparticles of 10 µm and 1 µm in diameter of the mode that is shown in Figure 4 (b1). As shown, the movements of these two particle species are distinct from each other (Figure 5). For both cases, an array of microparticles was released into the fluid domain within area −0.4≤x≤0.4 mm and −0.4≤y≤0.4 mm at t=0, and the trajectories of microparticles at three other time intervals, 0.1, 1, and 5 s, were presented. It can be seen that, under the effects of both acoustic radiation and streaming-induced drag forces (with the pressure and streaming magnitudes presented in Figure 4(1)), 10 µm particles were firstly rapidly moved to the pressure nodal line and then focused to the channel center by the acoustic radiation forces. On the pressure nodal line, the acoustic streaming-induced drag forces work against microparticle focusing. However, for the smaller 1 µm particles, their movements were dominated by the streaming-induced drag forces and, thus, the trajectories were following the acoustic streaming vortices.

## 5. Conclusions

In conclusion, we have shown here that with some modifications the LVM can also be applied to predict the outer streaming fields in fluid channels with curved surfaces. The variations of modes and outer streaming fields on cross-sections of a cylindrical cavity have been modelled by considering an easy-to-use 2D reduced-fluid model. When compared to the acoustophoretic motion of microparticles observed in experiments, the simulation of a full-device model might provide better prediction of the boundary vibrations and, thus, the standing wave and boundary-driven acoustic streaming patterns, due to the diversity of acoustic wave fields that could be generated in a real experimental device.

## Figures and Tables

**Table 1:** A summary of model parameters.

Parameters	Value	Units
Radius of cylindrical cavity	0.45	mm
Mesh size	9	μm
Dynamic viscosity of water	1.01	mPa·s
Density of water	999.6	kg/m^3^
Speed of sound in water	1481.4	m/s
